# NrtR Mediated Regulation of H1-T6SS in Pseudomonas aeruginosa

**DOI:** 10.1128/spectrum.01858-21

**Published:** 2022-02-23

**Authors:** Xinxin Zhang, Liwen Yin, Qi Liu, Dan Wang, Congjuan Xu, Xiaolei Pan, Fang Bai, Zhihui Cheng, Weihui Wu, Yongxin Jin

**Affiliations:** a State Key Laboratory of Medicinal Chemical Biology, Key Laboratory of Molecular Microbiology and Technology of the Ministry of Education, Department of Microbiology, College of Life Sciences, Nankai University, Tianjin, China; University of Maryland School of Pharmacy

**Keywords:** *P. aeruginosa*, H1-T6SS, NrtR, *rsmY*, *rsmZ*

## Abstract

NrtR is a Nudix-related transcriptional regulator that is distributed among diverse bacteria and plays an important role in modulating bacterial intracellular NAD homeostasis. Previously, we showed that NrtR influences the T3SS expression and pathogenesis of Pseudomonas aeruginosa and demonstrated that NrtR mediates T3SS regulation through the cAMP/Vfr pathway. In the present study, we found that mutation of the *nrtR* gene leads to upregulation of the Hcp secretion island-I type VI secretion system (H1-T6SS). Further analysis revealed that mutation of the *nrtR* gene results in upregulation of regulatory RNAs (RsmY/RsmZ) that are known to control the H1-T6SS by sequestration of RsmA or RsmN. Simultaneous deletion of *rsmY*/*rsmZ* reduced the expression of H1-T6SS in the Δ*nrtR* mutant. In addition, overexpression of either *rsmA* or *rsmN* in Δ*nrtR* decreased H1-T6SS expression. Chromatin immunoprecipitation (ChIP)-Seq and electrophoretic mobility shift assay (EMSA) analyses revealed that NrtR directly binds to the promoters of *rsmY*, *rsmZ* and *tssA1* (first gene of the H1-T6SS operon). Overall, the results from this study reveal the molecular details of NrtR-mediated regulation of H1-T6SS in P. aeruginosa.

**IMPORTANCE** NrtR is a Nudix-related transcriptional regulator and controls the NAD cofactor biosynthesis in bacteria. P. aeruginosa NrtR binds to the intergenic region between *nadD2* and pcnA to repress the expression of the two operons, therefore controlling the NAD biosynthesis. We have previously reported that NrtR controls T3SS expression via the cAMP/Vfr pathway in P. aeruginosa. However, the global regulatory function and direct binding targets of the NrtR remain elusive in P. aeruginosa. This study reveals novel direct regulatory targets of the NrtR in P. aeruginosa, elucidating the molecular mechanism of NrtR-mediated regulation of H1-T6SS.

## INTRODUCTION

Pseudomonas aeruginosa is a versatile opportunistic human pathogen that is responsible for a variety of infections in humans (1). The bacterium possesses several protein secretion systems that contribute to its pathogenesis and competitive advantage in the host environment (2). Among them, the type III secretion system (T3SS) is a critical virulence determinant that plays an important role in the interaction with the hosts during acute infections (3), while the type VI secretion system (T6SS) is a protein secretion machinery, deployed by P. aeruginosa to deliver effector proteins into neighboring eukaryotic or prokaryotic cells, that acts against both bacteria and hosts (4). In addition, T6SSs have also been reported to play roles in metal ion acquisition to improve the adaptation to environmental niches and interbacterial competition (5–8).

The P. aeruginosa genome encodes three different T6SS clusters, named H1-, H2-, and H3-T6SS (9). Among them, H1-T6SS displays an antibacterial activity and confers P. aeruginosa a growth advantage in competition over other T6SS^+^ bacteria inhabiting the same niche (10, 11). H1-T6SS comprises a hemolysin coregulated secretion island I (HSI-I) gene cluster and effectors scattered in its genome. The H1-T6SS cluster spans genes from PA0071 to PA0091 and encodes a set of components for the export apparatus (9). To date, eight H1-T6SS effector proteins have been identified in P. aeruginosa (4, 12).

The regulation of H1-T6SS expression can occur at the transcriptional, posttranscriptional, and posttranslational levels in P. aeruginosa (13). The quorum sensing regulator LasR and the 4-hydroxy-2-alkylquinoline transcriptional regulator MvfR negatively control the gene expression of H1-T6SS at the transcriptional level (14). RsmA and RsmN (RsmF), two CsrA family RNA-binding proteins, negatively regulate H1-T6SS at the posttranscriptional level (15–17). This control is determined by the availability of the free RsmA and RsmN proteins within bacterial cells, which are regulated via their interaction and sequestration by two small regulatory RNAs, RsmY and RsmZ (15, 17). In addition, H1-T6SS can be posttranslationally regulated by a threonine phosphorylation (TPP)-dependent or TPP-independent pathway (18, 19).

NrtR, a Nudix-related transcriptional regulator, is widely distributed across diverse bacterial species (20). It is composed of an N-terminal Nudix-like effector binding domain and a C-terminal DNA-binding winged helix-turn-helix (HTH) domain (20, 21). It acts as a transcriptional repressor via HTH domain-mediated binding to promoter regions of its target genes, while the Nudix domain specifically interacts with effector molecules to weaken the NrtR–DNA complex, resulting in derepression of target gene expression (20–22). It has been well documented that NrtR negatively regulates the *de novo* and salvage pathways of NAD cofactor biosynthesis to modulate intracellular NAD homeostasis (20–24). A previous study reported that NrtR influences the fitness and pathogenicity of the P. aeruginosa TBCF10839 strain (22).

Recently, we reported that P. aeruginosa NrtR controls the expression of T3SS through the cAMP/Vfr pathway (25). In this study, we demonstrate that NrtR regulates the expression of H1-T6SS genes by directly binding to the promoter of *tssA1*, as well as through the RsmY/RsmZ-RsmA/RsmN pathway. The presented work further reveals the multiplexed function of the NrtR in P. aeruginosa.

## RESULTS

### Mutation of *nrtR* increases the expression of H1-T6SS genes in P. aeruginosa.

**FIG 1:**
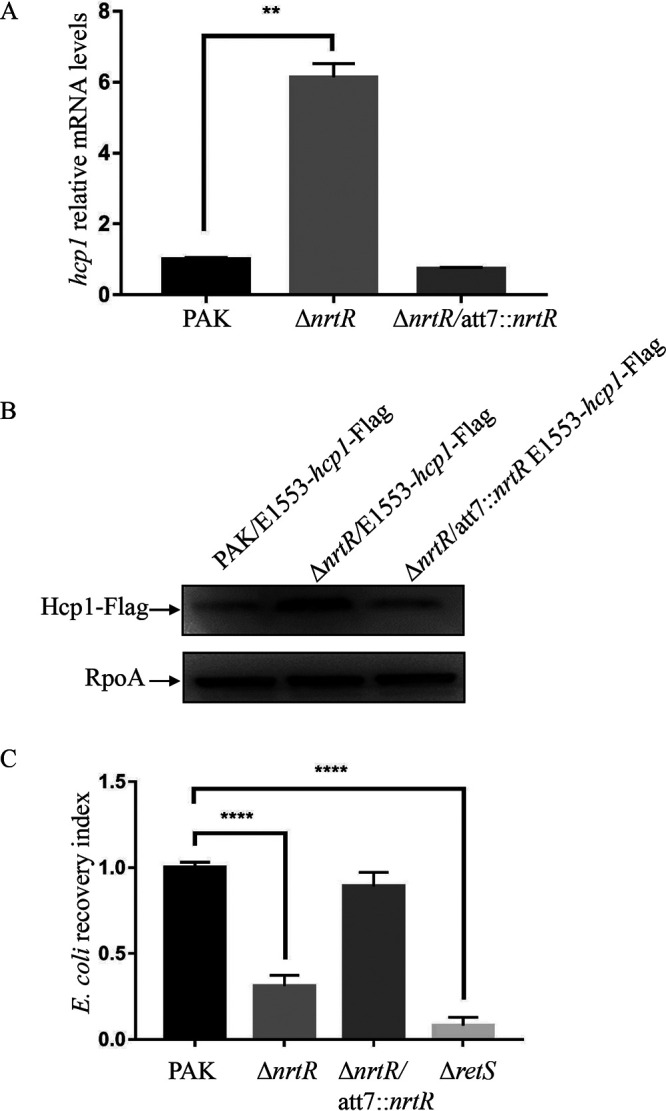
Hcp1 was upregulated in the Δ*nrtR* mutant. (A) The relative *hcp1* mRNA levels in PAK, Δ*nrtR*, and Δ*nrtR*/att7::*nrtR*. Total RNA was isolated from bacteria at an OD_600_ of 1.0, and *hcp1* mRNA levels were examined by real-time qPCR using *rpsL* as an internal control. **, *P < *0.01, by Student's *t* test. (B) Bacteria containing an *hcp1*-Flag driven by its native promoter were grown to an OD_600_ of 1.0 in LB medium. Proteins from an equivalent number of P. aeruginosa cells of the indicated strains were separated on 12% SDS-PAGE and probed with an antibody against Flag or RpoA. (C) Competition assay between P. aeruginosa and E. coli. The indicated P. aeruginosa strains and E. coli were mixed at a 5:1 ratio, coincubated for 24 h at 25°C, resuspended in LB, and plated on LB agar plates with tetracycline. The E. coli recovery index represents the bacterial number of recovered DH5α/pDN19 with the number of DH5α/pDN19 in competition with PAK as 1.0. Bars represent the means from three experiments, and error bars indicate the standard deviation. ****, *P < *0.0001, by Student's *t* test.

To determine the global regulatory role of *nrtR* in P. aeruginosa, RNAseq analysis was carried out to compare the global transcriptomes between the wild-type strain PAK and its Δ*nrtR* mutant derivative. As revealed by the transcriptomic analyses, most genes of the H1-T6SS operon and its scattered effectors/immunity proteins (*tse1*/*tsi1*, *tse3*/*tsi3*, *tse4*, *tse5*, and *tse6*/*tsi6*) were upregulated in the Δ*nrtR* mutant (Table 1; complete list shown in Table S3 in the supplemental material). In order to understand the relationship between the *nrtR* and H1-T6SS genes, we used real-time qPCR to verify the mRNA levels of the Hcp1-encoding gene, the hallmark of the H1-T6SS in P. aeruginosa. As shown in Fig. 1, the mRNA level of *hcp1* was upregulated 6.1-fold in the Δ*nrtR* mutant and restored to wild-type PAK level by complementation with a wild-type *nrtR* gene. To verify the increased expression level of Hcp1, a C-terminal Flag-tagged Hcp1 driven by its native promoter was further introduced into the PAK and Δ*nrtR* mutant. Consistent with the real-time qPCR results, the Hcp1 protein level was elevated in the Δ*nrtR* mutant and restored to that in PAK by the complementation with *nrtR* (Fig. 1B).

**TABLE 1:** mRNA levels of genes related to H1-T6SS in Δ*nrtR* compared to those in PAK identified via RNAseq (57)

Gene ID	Gene name	Fold change (Δ*nrtR*/PAK)	Description
PA0070	*tagQ1*	5.2	TagQ1
PA0072	*tagS1*	5.7	TagS1
PA0073	*tagT1*	5.3	TagT1
PA0074	*ppkA*	5.7	Serine/threonine protein kinase PpkA
PA0075	*pppA*	7.5	PppA
PA0076	*tagF1*	6.1	TagF1
PA0077	*icmF1*	10.5	IcmF1
PA0078	*tssL1*	11.4	TssL1
PA0079	*tssK1*	11.8	TssK1
PA0080	*tssJ1*	9.1	TssJ1
PA0082	*tssA1*	9.6	TssA1
PA0083	*tssB1*	20.8	TssB1
PA0084	*tssC1*	18.0	TssC1
PA0085	*hcp1*	21.1	Hcp1
PA0086	*tagJ1*	21.4	TagJ1
PA0087	*tssE1*	20.7	TssE1
PA0088	*tssF1*	29.5	TssF1
PA0089	*tssG1*	32.6	TssG1
PA0090	*clpV1*	17.5	ClpV1
PA0091	*vgrG1*	17.4	VgrG1
PA0092	*tsi6*	5.8	Tsi6
PA0093	*tse6*	15.4	Tse6
PA0096^*a*^		8.9	Hypothetical protein
PA0097^*a*^		5.0	Hypothetical protein
PA1844	*tse1*	9.2	Tse1
PA1845	*tsi1*	9.5	Tsi1
PA2684	*tse5*	6.0	Tse5
PA2774	*tse4*	7.7	Tse4
PA3484	*tse3*	9.0	Tse3
PA3485	*tsi3*	7.2	Tsi3

a Genes located in the *vgrG1b* cluster (57).

Since H1-T6SS plays an important role in the fitness advantage of P. aeruginosa in competition with other bacteria (26), the functional connection between NrtR and H1-T6SS prompted us to determine the role of NrtR in the interspecies competition. Accordingly, we performed interbacterial growth competition experiments between P. aeruginosa and E. coli. Competition assays were conducted for 24 h at 25°C, using the P. aeruginosa *retS* mutant, which shows a constitutively active H1-T6SS (9) as a positive control. As shown in Fig. 1C, the E. coli recovery index was significantly decreased when mixed with the Δ*nrtR* mutant compared to that with wild-type PAK, and the recovery index was restored by complementation with the *nrtR* gene. These results demonstrated that the expression of H1-T6SS is upregulated in the Δ*nrtR* mutant.

### Upregulation of *rsmY*/*rsmZ* contributes to the increased H1-T6SS in the Δ*nrtR* mutant.

Since NrtR controls the expression of T3SS through the cAMP/Vfr pathway (25), we wanted to know if the increased H1-T6SS in the Δ*nrtR* mutant was due to the decreased intracellular cAMP levels. To test it, we examined the expression of H1-T6SS in the Δ*cyaA*Δ*cyaB* and Δ*vfr* mutants, which also showed reduced intracellular cAMP amounts (25). As real-time qPCR results shown in Fig. S1A, both *hcp1* and *tssA1* displayed decreased mRNA levels in Δ*cyaA*Δ*cyaB* and Δ*vfr* mutants compared to that in the wild-type PAK strain. These data indicate that upregulation of H1-T6SS was not due to the decreased cAMP levels in the Δ*nrtR* mutant.

In P. aeruginosa, the expression of H1-T6SS was shown to be controlled by intracellular c-di-GMP (27), which is linked to the cAMP signaling pathways (28, 29). We previously reported that the cAMP level was significantly decreased in the Δ*nrtR* mutant (25); thus, we wanted to examine whether *nrtR* affects the intracellular c-di-GMP level. The expression of surface adhesion CdrA is known to be regulated by intracellular c-di-GMP; thus, its expression level has been utilized as an indicator of intracellular c-di-GMP levels (30, 31). Accordingly, real-time qPCR was carried out to compare the relative mRNA levels of *cdrA* between PAK and the Δ*nrtR* mutant derivative. Compared to that in PAK, no significant difference in *cdrA* mRNA level was observed in Δ*nrtR* (Fig. S1B), suggesting that NrtR has no influence on the P. aeruginosa intracellular c-di-GMP level.

**FIG 2:**
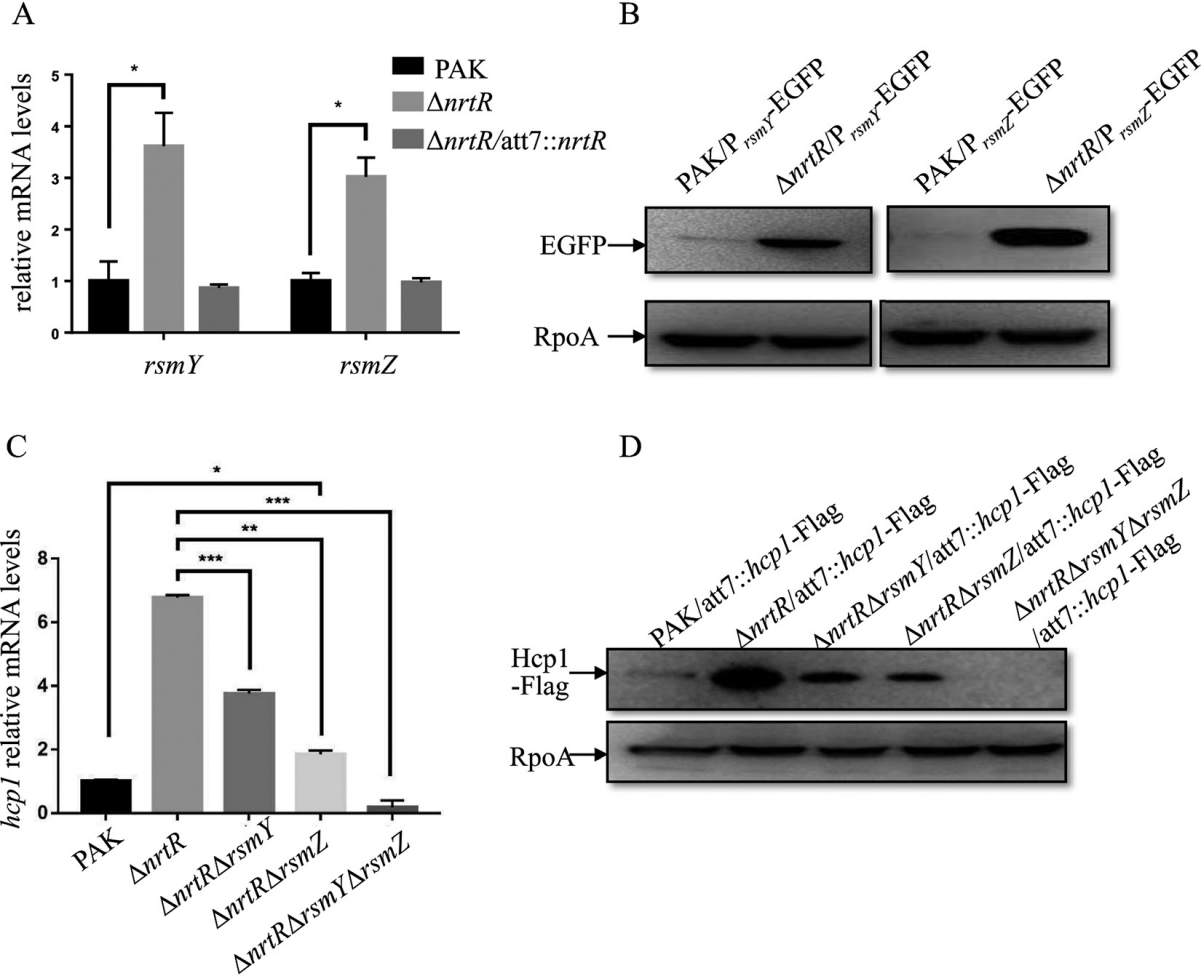
Upregulation of RsmY/RsmZ contributes to the increased H1-T6SS in the Δ*nrtR* mutant. (A, C) The relative RNA levels of *rsmY*, *rsmZ* (A), and *hcp1* (C) in the indicated strains. Total RNA was isolated from bacteria at an OD_600_ of 1.0, and the relative RNA levels of *rsmY*, *rsmZ*, and *hcp1* were examined by real-time qPCR using *rpsL* as an internal control. *, *P < *0.05; **, *P < *0.01; ***, *P < *0.001 by Student's *t* test. (B, D) Bacteria containing an *egfp* gene driven by the promoter of *rsmY* or *rsmZ* (B) or bacteria integrated with an *hcp1*-Flag driven by its native promoter (D) were grown to an OD_600_ of 1.0 in LB medium. Proteins from an equivalent number of P. aeruginosa cells of the indicated strains were separated on a 12% SDS-PAGE and probed with an antibody against EGFP, Flag, or RpoA.

In P. aeruginosa, the small RNAs RsmY and RsmZ are known to regulate the expression of H1-T6SS (32). In order to test whether *nrtR* repressed H1-T6SS through RsmY and RsmZ, we compared the levels of these sRNAs between PAK and the Δ*nrtR* mutant. Indeed, compared to wild-type PAK or Δ*nrtR* with complementation, the *rsmY* and *rsmZ* levels in the Δ*nrtR* mutant were 3.6- and 3.0-fold higher, respectively (Fig. 2A). To confirm the increased expression of *rsmY* and *rsmZ*, transcriptional fusion plasmids of P*_rsmY_* and P*_rsmZ_* to an *egfp* gene (P*_rsmY_*-EGFP and P*_rsmZ_*-EGFP) were constructed and introduced into the above bacterial strains. Consistent with the differential mRNA levels, the EGFP protein amounts were higher in the Δ*nrtR* mutant background than that in PAK (Fig. 2B). These results indicated that RsmY and RsmZ are upregulated in the Δ*nrtR* mutant.

To test the role of RsmY and RsmZ in the regulation of H1-T6SS mediated by NrtR, we constructed Δ*nrtR*Δ*rsmY* and Δ*nrtR*Δ*rsmZ* double mutants, as well as a Δ*nrtR*Δ*rsmY*Δ*rsmZ* triple mutant, and examined their expression of H1-T6SS. As shown in Fig. 2C and D, individual deletion of *rsmY* or *rsmZ* in Δ*nrtR* decreased the expression of *hcp1*, but not to the level of the wild-type PAK strain. However, simultaneous deletion of *rsmY* and *rsmZ* in the Δ*nrtR* background resulted in the expression of *hcp1* below that of the wild-type PAK strain. These data together suggest that NrtR represses H1-T6SS through the small RNAs RsmY and RsmZ.

### Overexpression of *rsmA*/*rsmN* in the Δ*nrtR* mutant restores the expression of H1-T6SS.

**FIG 3:**
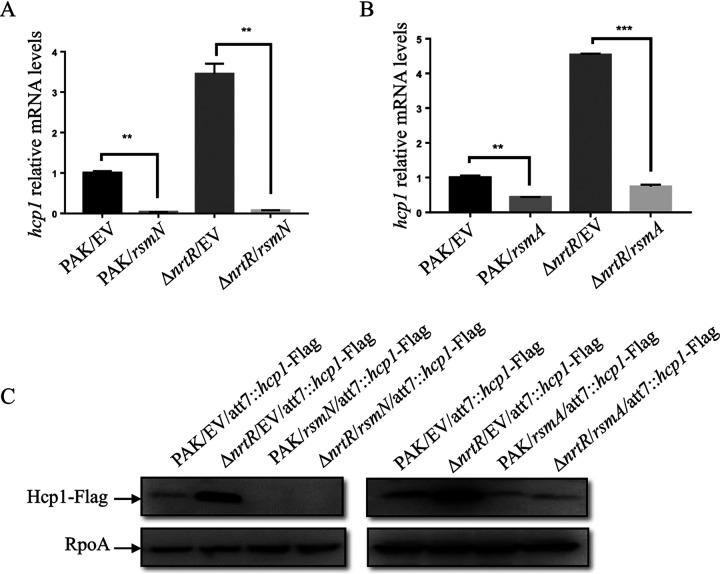
Overexpression of *rsmA*/*rsmN* in the Δ*nrtR* mutant restored the expression of H1-T6SS. (A, B) The relative *hcp1* mRNA levels in PAK and Δ*nrtR* containing pUCP20 (empty vector, EV), pUCP20-*rsmN*, or pUCP20-*rsmA*. Total RNA was isolated from bacteria at an OD_600_ of 1.0, and *hcp1* mRNA levels were examined by real-time qPCR using *rpsL* as an internal control. **, *P < *0.01; ***, *P < *0.001, by Student's *t* test. (C) PAK and Δ*nrtR* integrated with an *hcp1*-Flag driven by its native promoter containing pUCP20 (empty vector, EV), pUCP20-*rsmN* (*rsmN*), or pUCP20-*rsmA* (*rsmA*) were grown to an OD_600_ of 1.0 in LB medium. Proteins from an equivalent number of P. aeruginosa cells of the indicated strains were separated on a 12% SDS-PAGE and probed with an antibody against Flag or RpoA.

Since the major role of RsmY and RsmZ is to sequester and lower free RsmA and RsmN, two CsrA family RNA binding proteins, we investigated whether ectopic expression of *rsmA* or *rsmN* could restore H1-T6SS expression in the Δ*nrtR* mutant. First, real-time qPCR was performed to determine the transcriptional level of *hcp1*. As expected, overexpression of either *rsmA* or *rsmN* decreased the relative mRNA levels of *hcp1* in the Δ*nrtR* mutant (Fig. 3A and B). Furthermore, a Flag-tagged *hcp1* driven by its native promoter (33) was integrated into the chromosomes of PAK and its Δ*nrtR* derivative. Western blot assay was carried out to examine the expression of Hcp1. Consistent with the real-time qPCR results, overexpression of either *rsmA* or *rsmN* decreased the Hcp1-Flag amounts in Δ*nrtR* (Fig. 3C). These results indicate that NrtR influences RsmA/RsmN-mediated regulation of the H1-T6SS.

### NrtR binds directly to the promoter of *rsmY*/*rsmZ*.

To understand the NrtR-mediated *rsmY*/*rsmZ* regulation, we further examined whether NrtR affects the expression of known regulatory genes upstream of the *rsmY*/*rsmZ* genes, such as *gacA*, *gacS*, *ladS*, *retS*, *algR*, and *amrZ* (27, 32, 34–36). Interestingly, the mRNA levels of all of these regulatory genes were not altered in the Δ*nrtR* mutant based on the RNAseq analysis and real-time qPCR (Fig. S1B).

**FIG 4:**
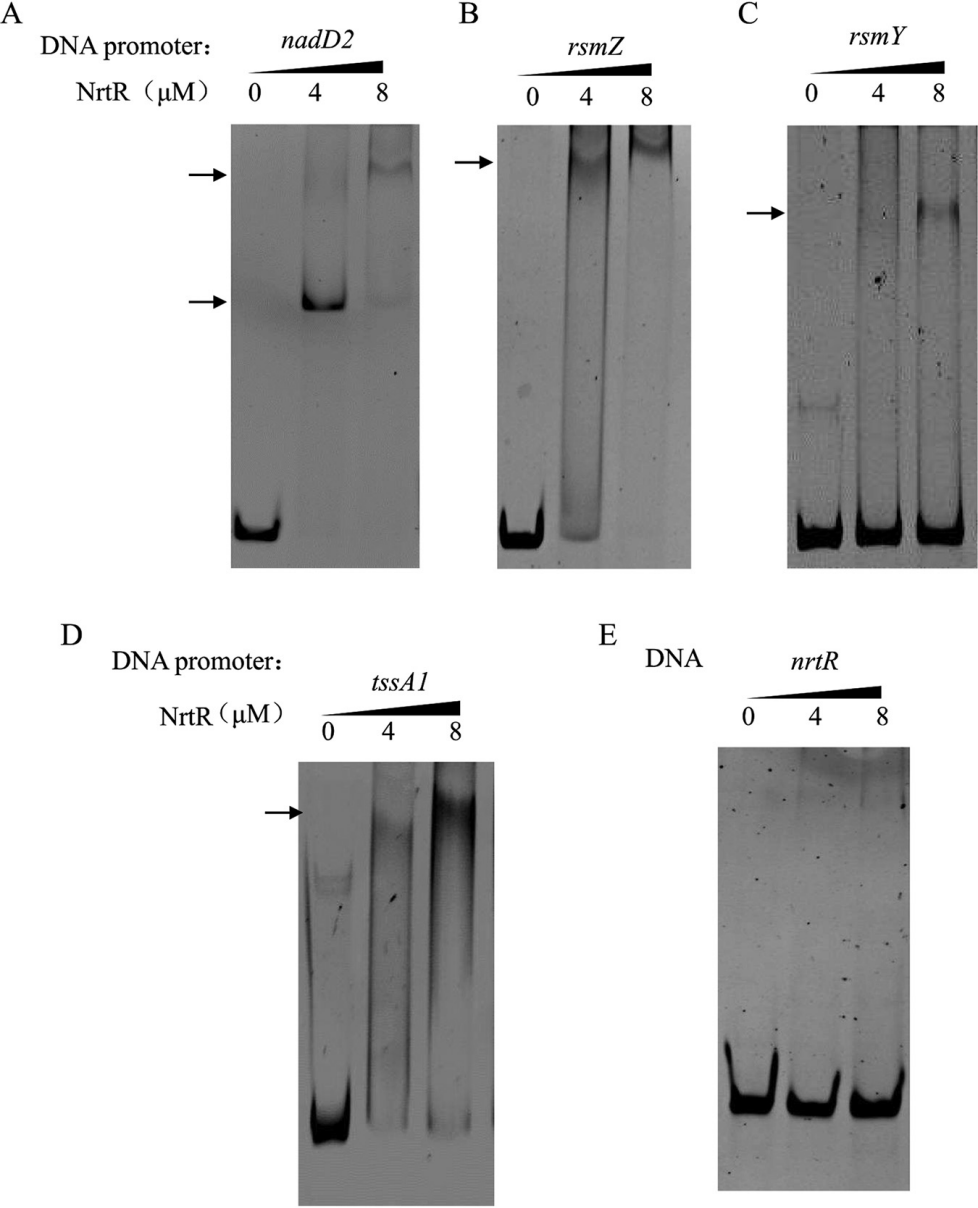
NrtR binds directly to the promoter of *rsmY*, *rsmZ*, or *tssA1*. Binding of NrtR to the DNA fragment corresponding to the promoter regions of *nadD2* (A), *rsmZ* (B), *rsmY* (C), *tssA1* (D), or the inner region of *nrtR* (E) was determined by EMSA. Increasing amounts of the purified His-NrtR were incubated with 40 ng of the indicated DNA fragments. The mixtures were electrophoresed on an 8% native PAGE gel, and the bands were visualized under UV light following ethidium bromide staining. Data represent results from three independent experiments. The arrows indicated the DNA-protein complex.

To further elucidate the molecular mechanism of *rsmY*/*rsmZ* regulation by NrtR, a ChIP-seq assay was performed. First, an N-terminal Flag-tagged *nrtR* expression plasmid pUCP24-*nrtR* was constructed, which was able to restore the expression of H1-T6SS in the Δ*nrtR* mutant (data not shown). Then, ChIP-seq was carried out in the Δ*nrtR* mutant harboring the expressing plasmid. The DNA binding loci and enrichment folds are displayed in Table S4. Consistent with a previous report that NrtR binds an intergenic region between *nadD2* and pcnA (22), the intergenic region was enriched by 2.21-fold (Table 2). Of note, the potential binding targets of NrtR included sequences upstream of the *rsmY* and *rsmZ* genes (Table 2). EMSA was further performed to validate the binding of NrtR to the candidate promoter regions. A His-tagged NrtR was overexpressed in pET28a and purified from E. coli. Binding of the His-NrtR to the *nadD2* promoter was used as a positive control. As shown in Fig. 4, upon incubation with His-NrtR, shifted bands were detected for DNA fragments corresponding to promoter regions of *nadD2*, *rsmY*, and *rsmZ*, but not for the negative control DNA fragment (*nrtR*), indicating that NrtR binds directly to the promoter regions of *rsmY* and *rsmZ*.

**TABLE 2:** Potential NrtR regulated genes identified via ChIP-seq analysis

Gene ID of PAK	Gene ID of PAO1	Summit in PAK	Fold enrichment	Gene name
PAK_00300	PA0082	332033	1.55	*tssA1*
PAK_00742	PA0527.1	806568	1.47	*rsmY*
PAK_01565	PA3621.1	1674747	1.43	*rsmZ*
PAK_05420	PA4917	5879334	2.21	*nadD2*

### NrtR directly binds to the *tssA1* promoter and represses *tssA1* expression.

**FIG 5:**
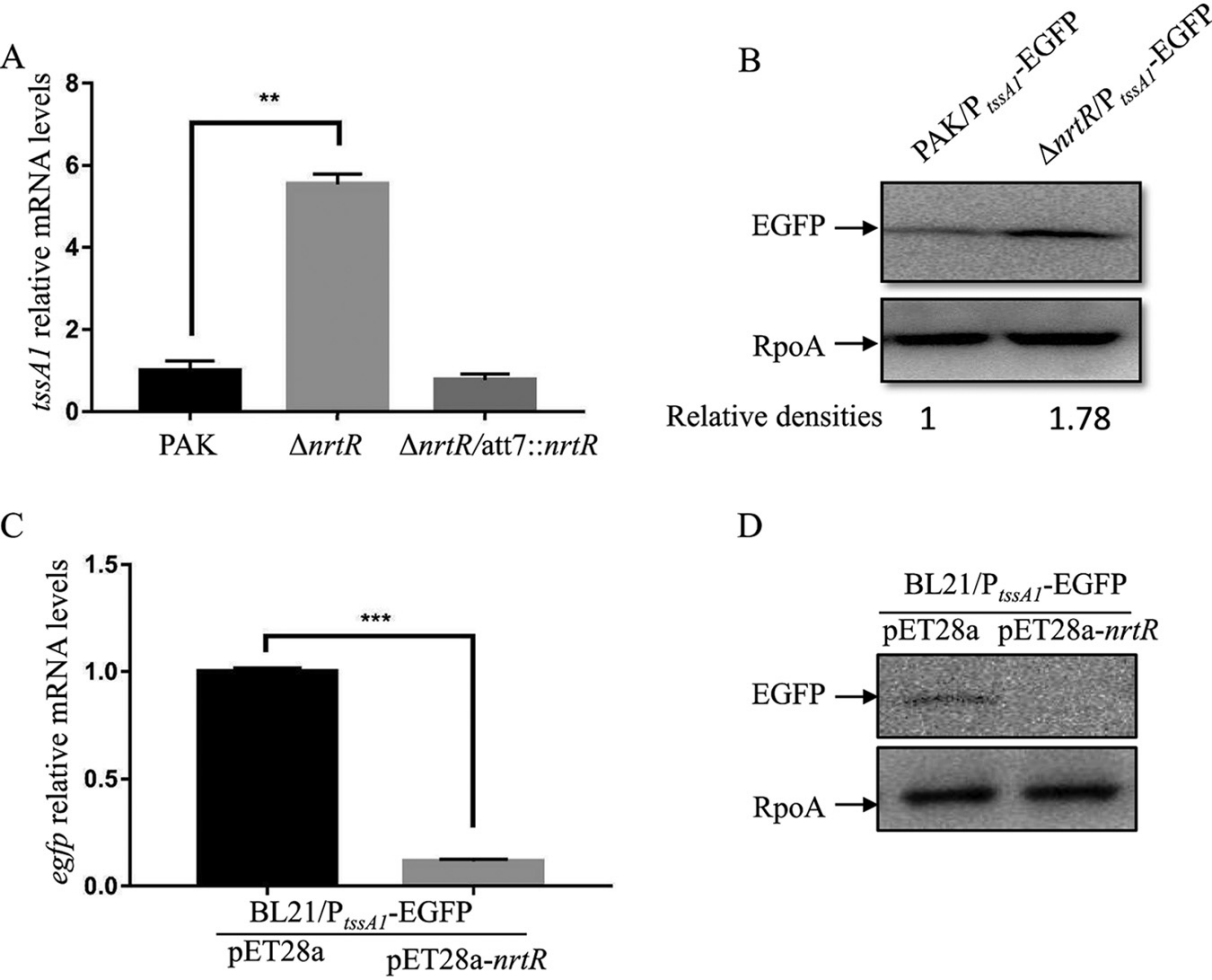
*tssA1* was repressed by NrtR directly. (A, C) Real-time qPCR assay. (A) The relative mRNA levels of *tssA1* in the PAK, Δ*nrtR*, and Δ*nrtR* complement strains. (C) The relative mRNA levels of *egfp* in BL21 containing both P*_tssA1_*-EGFP (*egfp* gene driven by *tssA1* promoter) and pET28a or pET28a-*nrtR* plasmids. Total RNA was isolated from bacteria at an OD_600_ of 1.0, and the indicated mRNA levels were examined by real-time qPCR using *rpsL* as an internal control. **, *P < *0.01; ***, *P < *0.001, by Student's *t* test. (B, D) Western blot assay. PAK and Δ*nrtR* containing a P*_tssA1_*-EGFP plasmid (B) or BL21 containing both P*_tssA1_*-EGFP and pET28a or pET28a-*nrtR* plasmids (D) were grown to an OD_600_ of 1.0 in LB medium. Proteins from an equivalent number of bacterial cells of the indicated strains were separated on a 12% SDS-PAGE and probed with an antibody against EGFP or RpoA. Relative densities represent the density of EGFP/density of RpoA with the first lane as 1.

In addition to *rsmY* and *rsmZ*, our ChIP-seq data revealed that the promoter of *tssA1*, the first gene of the H1-T6SS operon, is also a potential binding target of NrtR (Table 2). Further supporting this, the *tssA1* transcriptional level displayed a 9.6-fold increase in the Δ*nrtR* mutant according to the RNAseq analysis (Table 1). Therefore, it is possible that NrtR directly binds to the *tssA1* promoter and represses its expression. To test this possibility, we first validated the increased expression of *tssA1* in Δ*nrtR* using real-time qPCR. As shown in Fig. 5A, agreeing with the RNAseq result, the relative mRNA level of *tssA1* was significantly increased in the Δ*nrtR* mutant, which could be restored to that of the wild-type PAK strain by complementation with *nrtR*. A transcriptional fusion plasmid of P*_tssA1_* and an *egfp* gene (P*_tssA1_*-EGFP) were generated and introduced into PAK and the Δ*nrtR* mutant. Consistent with the mRNA level, the protein level of EGFP driven by P*_tssA1_* was significantly higher in the Δ*nrtR* mutant background (Fig. 5B). To further verify the direct repression of *tssA1* expression by NrtR, *nrtR* was cloned into pET28a and cointroduced into the E. coli BL21 strain with the P*_tssA1_*-EGFP plasmid. Real-time qPCR and Western blot were carried out to determine the expression level of *egfp* driven by the promoter of *tssA1*. As shown in Fig. 5C and D, compared to the empty vector pET28a, the expression of *nrtR* decreased the expression of *egfp* in the BL21 strain, which indicates a direct repression of the *tssA1* promoter by NrtR.

To further validate the direct binding of NrtR to the promoter region of *tssA1*, we performed EMSA using fragments corresponding to the promoter region of *tssA1*. Similar to the promoters of *rsmY* and *rsmZ*, upon incubation with NrtR, a retarded band was detected for the *tssA1* promoter region, but not for the DNA fragment of *nrtR* (Fig. 4), indicating that NrtR binds to the promoter region of the H1-T6SS operon specifically.

## DISCUSSION

**FIG 6:**
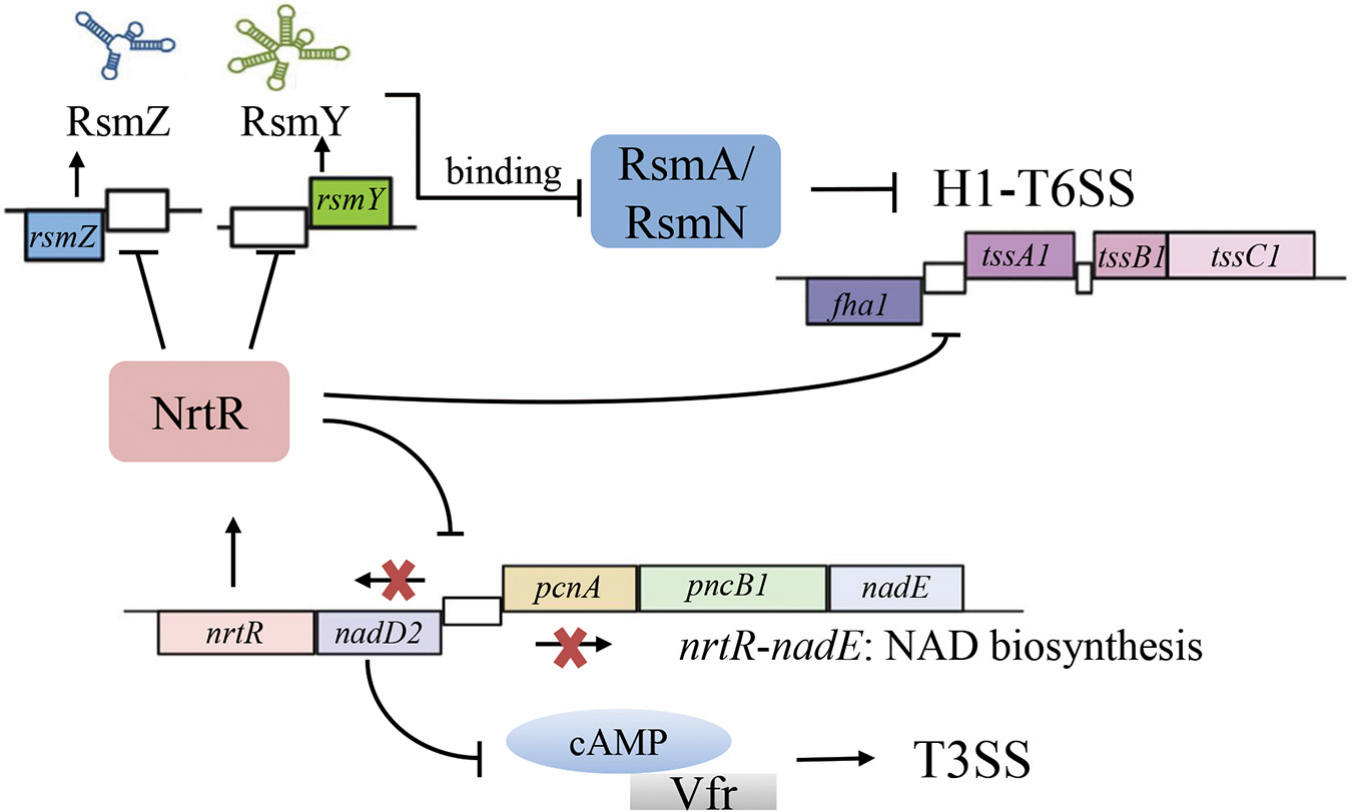
Proposed model of NrtR-mediated regulation in P. aeruginosa.

NrtR is a transcriptional repressor of NAD biosynthesis in P. aeruginosa (22) that can bind to the intergenic region between *nadD2* and pcnA directly and thereby represses transcription of the divergently transcribed operons *nadD2*-*nrtR* and pcnA-*nadE* (22). Previously, we reported that NrtR affects intracellular cAMP levels and is required for the expression of T3SS as well as pathogenesis in P. aeruginosa (25). Here, we demonstrate that P. aeruginosa NrtR represses the expression of H1-T6SS genes. Further studies revealed that NrtR controls H1-T6SS directly by binding to the promoter of *tssA1* and indirectly through the RsmY/RsmZ-RsmA/RsmN pathway by directly regulating the transcription of *rsmY* and *rsmZ*. Combining all of the above, a model has been proposed for the *nrtR* gene function in P. aeruginosa (Fig. 6).

It has been demonstrated that the transcriptional regulator AmrZ activates expression of H1-T6SS in P. aeruginosa (37). Using real-time qPCR, we found that expression of *amrZ* was not affected in the Δ*nrtR* mutant (Fig. S1B), suggesting that *amrZ* was not involved in the NrtR-mediated regulation of H1-T6SS. Of note, AmrZ binds directly to the *tssA1* upstream region (37). The putative binding sequence of AmrZ is CACAACGCCACTA, which locates −169∼–156 bp upstream of the start codon of *tssA1* (37). In this study, we found that NrtR directly binds to the *tssA1* upstream region to repress expression of the H1-T6SS. When using the 198 bp DNA fragment immediate upstream of the AmrZ binding site as probe, we also observed the direct binding by NrtR in our EMSA experiment (data not shown). Thus, we assume that the binding regions of NrtR and AmrZ in the *tssA1* upstream region are not overlapped. However, the antagonistic control of H1-T6SS by these two regulators remains elusive and warrants further studies.

In addition to the multiplexed function of NrtR in P. aeruginosa, the NrtR-type transcription factor AraR has been reported to control L-arabinose catabolism and utilization of arabinose-containing polysaccharides in Bacteroides thetaiotaomicron (38). NrtR has also been shown to control the biofilm forming capacity and the pathogenesis of the zoonotic pathogen Streptococcus suis in a mouse infection model (23). In view of the function of *nrtR* in the biofilm forming in Streptococcus suis, we also compared the biofilm formation between PAK and the Δ*nrtR* mutant. Our preliminary results demonstrated a reduction of biofilm formation in the Δ*nrtR* mutant (Fig. S2A). Currently, we are making efforts to explore the mechanism of *nrtR* mediated regulation on biofilm formation in P. aeruginosa.

NrtR controls the NAD cofactor biosynthesis to modulate NAD homeostasis in P. aeruginosa (22). In addition, NrtR regulates H1-T6SS of P. aeruginosa. Interestingly, the H1-T6SS substrate Tse2 has been suggested to be a NAD-dependent toxin (39). Another H1-T6SS effector, Tse6, acts on target cells by degrading the dinucleotides NAD^+^ and NADP^+^ in P. aeruginosa (40). However, the relevance of these toxins in connection with the role of NrtR in NAD biogenesis in P. aeruginosa remains elusive and warrants further studies.

P. aeruginosa harbors three different T6SS clusters. We found that the *nrtR* mutation leads to upregulation of H1-T6SS, but has no effect on the expression of H2-T6SS and H3-T6SS (Fig. S2B). Of note, using a Genechip analysis, a recent study revealed that the transcriptional levels of *hcp1* and *tssB1*, two components of H1-T6SS, were upregulated in a TBCF10839*ntrR*::Tn strain. In addition, *hsiB3* and *hsiC3*, two components of H3-T6SS, also displayed a 7-fold increase at the transcriptional level (22). Such a differential effect on H3-T6SS expression might be due to the difference in strain backgrounds used in these two studies. TBCF10839 is a mucoid P. aeruginosa strain isolated from a cystic fibrosis patient with high-level production of pyocyanin, as well as quorum sensing signal molecules PQS and *N*-acylhomoserine lactones (41). In contrast, PAK is a laboratory model strain with nonmucoid phenotype.

Our real-time qPCR and promoter fusion assays revealed an increased expression of *rsmY* and *rsmZ* in the Δ*nrtR* mutant (Fig. 2). However, this upregulation was not observed in our RNAseq analysis. It is possible that the small RNAs RsmY and RsmZ might have low recovery during the process of library generation.

The expression of RsmY/Z is controlled by a complex regulatory network. The two-component signal transduction system GacS-GacA positively regulates the transcription of *rsmY* and *rsmZ* by direct binding of the regulator GacA to sequences upstream of these genes (32). Hybrid sensor kinases LadS and RetS, and the histidine phosphotransfer protein HptB intersect with GacA to modulate the expression of *rsmY* or/and *rsmZ* (34, 42–44). The magnesium transporter MgtE also activates *rsmY* and *rsmZ* transcription through the GacS-GacA two-component system (45). NarL, an Anr-regulated response regulator, represses the transcription of *rsmY* and *rsmZ* by directly binding to their promoters (46). MvaT and MvaU, the H-NS family of DNA binding proteins, function as transcriptional repressors of *rsmZ* by binding to its promoter (32). BswR, a xenobiotic response transcriptional regulator, modulates *rsmZ* transcription by binding to its promoter and counters the repression by MvaT (47). Transcription of both *rsmY* and *rsmZ* is also positively controlled by RsmA via an unknown mechanism (48). Recently, it has been reported that small RNA 179 expression stimulates *rsmY* transcription (49). In addition, the levels of RsmY/Z are modulated at the posttranscriptional level. The interaction between PNPase and RsmY/Z controls the stability of these sRNAs (50). Hfq interacts with RsmY and protects it from cleavage by RNase E (51, 52). Here, we identified NrtR as a repressor of the *rsmY*/*Z*, which represses the transcription of *rsmY*/*Z* by directly binding to the upstream regions of these genes.

In summary, we identified NrtR as a repressor of the H1-T6SS in P. aeruginosa and revealed its repressing role on the H1-T6SS by both direct and indirect effects via the regulatory RNAs RsmY and RsmZ.

## MATERIALS AND METHODS

### Bacterial strains, plasmids, and growth conditions.

Bacterial strains and plasmids used in this study are shown in Table S1. Bacterial cells were grown in Luria–Bertani (LB) medium, which contained 5 g/L NaCl, 5 g/L yeast extract, and 10 g/L tryptone or on LB agar plates (supplemented with 15 g/L agar) at 37°C. When needed, the medium was supplemented with appropriate antibiotics at the following concentrations: for P. aeruginosa: tetracycline 50 μg/mL, gentamicin 50 μg/mL, carbenicillin 150 μg/mL; for E. coli: tetracycline 10 μg/mL, kanamycin 25 μg/mL, gentamicin 10 μg/mL, and ampicillin 100 μg/mL.

### Construction of plasmids and bacterial strains.

To delete the *rsmY* gene, the deletion plasmid pEX18-*rsmY* (33) was electroporated into the E. coli S17-1 strain, followed by conjugal transfer to P. aeruginosa strains. The *rsmY* deletion was carried out by homologous recombination in the Δ*nrtR* mutant followed by selection for single crossover and then double crossover, as previously described (53). The target *rsmY* deletion mutant was verified by PCR amplification (primers in Table S2). The *rsmZ* gene deletion in Δ*nrtR* and Δ*nrtR*Δ*rsmY* was carried out by similar methods with the previously constructed plasmid pEX18-*rsmZ* (33).

Plasmid pUCP20-*rsmA* was constructed by PCR amplification of the *rsmA* open reading frame (ORF) and its putative Shine-Dalgarno (SD) sequence from PAK genomic DNA using primers pUCP-*rsmA*F and pUCP-*rsmA*R. The PCR products were digested with *Eco*RI-*Hin*dIII and then cloned into the pUCP20 plasmid. pUCP20-*rsmN*, pUCP24-*nrtR*, and pET28a-*nrtR* were constructed by a similar strategy. E1553-*hcp1*-Flag was generated by PCR amplification of the C-terminal Flag-tagged *hcp1* gene and its promoter region from PAK genomic DNA with specific primers (Table S2). The PCR products were digested with *Xba*I-*Hin*dIII and then cloned into the promoterless pUCP20, whose promoter was removed by replacing nucleotides (GCTTTC) at sites 1813–1818 with the *Eco*RI digestive site (GAATTC) followed by *Eco*RI digestion and self-ligation (54).

To generate the construct of P*_rsmY_*-EGFP, the promoter region of *rsmY* was amplified by PCR from PAK genomic DNA and inserted upstream of the promoterless-*egfp* gene in p19-EGFP, which was constructed by cloning the *egfp* gene into the *Bam*HI-*Hin*dIII sites of pDN19*lacZΩ*. The constructs of P*_rsmZ_*-EGFP and P*_tssA1_*-EGFP were made with the same procedure.

To generate the Δ*nrtR*/att7::*hcp1*-Flag strain, pUC18T-mini-Tn7T-*hcp1*-Flag, along with the helper plasmid pTNS3, was electroporated into the Δ*nrtR* mutant. Insertion of the P*_hcp1_*-*hcp1*-Flag fragment into the chromosome was verified by PCR amplification with primers P*_glmS_*-down and P*_Tn7R_* (Table S2). Strain PAK/att7::*hcp1* was generated with similar strategy.

### Western blot.

A single bacterial colony was inoculated into LB medium, cultivated overnight, diluted 1:50 into 3 mL fresh LB medium, and then grown to an OD_600_ of 1.0 with shaking at 200 rpm. Proteins from an equivalent number of bacterial cells were mixed with loading buffer, boiled for 10 min at 99°C, and then loaded onto and separated by a 12% sodium dodecyl sulfate-polyacrylamide (SDS-PAGE) gel. Proteins were then transferred onto a polyvinylidene difluoride (PVDF) membrane and probed with an antibody against EGFP (GeneTex), Flag (Sigma), or RpoA (RNAP, Abcam) for 1–2 h at room temperature or overnight at 4°C. The signals were detected using an ECL Plus kit (Millipore) and visualized in a Bio-Rad molecular imager (ChemiDocXRS).

### RNA purification, real-time qPCR, and RNAseq analysis.

Overnight cultures of bacteria were diluted into fresh LB medium (1:50 dilution) and cultivated to an OD_600_ of 1.0 at 37°C. Total RNA was isolated using an RNAprep Pure Kit (Cell/Bacteria, Tiangen Biotec, Beijing, China). cDNA was synthesized from 1 μg of RNA using PrimeScript Reverse Transcriptase with random primers (TaKaRa, Dalian, China). For real-time qPCR, cDNA was mixed with specified primers (Table S2) and SYBR Premix Ex TaqTM II (TaKaRa, Dalian, China). The reactions were conducted using a CFX Connect Real-Time system (Bio-Rad). The 30S ribosomal protein gene *rpsL* was utilized as an internal control. The gene expression level was examined using the 2^-ΔΔCt^ method. For RNAseq analysis, the total RNA was sent to GENEWIZ (Suzhou, China) to conduct the sequencing and analysis as described before (55).

### Bacterial competition assay.

Overnight cultures of P. aeruginosa strains and E. coli DH5α/pDN19 were diluted 1:50 into 3 mL fresh LB medium and grown to an OD_600_ of 1.0. The cells of P. aeruginosa and DH5α/pDN19 were collected by centrifugation, washed twice with LB medium, and mixed at a ratio of 5:1. 50 μl of this mixture was spotted onto a nitrocellulose membrane with 0.22 μm pores (Solarbio, Beijing, China) on an LB agar plate. After drying, the mixtures were incubated for 24 h at 25°C. Then, bacterial cells on the nitrocellulose membrane were resuspended in 1 mL LB, serially diluted in LB medium, and plated onto LB agar plates with 50 μg/mL tetracycline to enumerate the CFU of DH5α/pDN19. The E. coli recovery index represents the bacterial number of recovered DH5α/pDN19 with the number of DH5α/pDN19 cells in competition with PAK as 1.0.

### Expression and purification of recombinant NrtR protein.

To express recombinant NrtR with an N-terminal His-tag, the *nrtR* gene was cloned into pET28a to form pET28a-*nrtR*. E. coli strain BL21(DE3) was transformed with the construct and grown in LB at 37°C to an OD_600_ of 0.6. Then, 0.1 mM isopropyl-β-D-1-thiogalactopyranoside (IPTG) was added to induce the expression of NrtR. After an additional 4 h of cultivation, cells were harvested from 100 mL bacterial culture and frozen overnight at −20°C. The frozen cells were resuspended in 5 mL lysis buffer A (46.6 mM Na_2_HPO_4_, 3.4 mM NaH_2_PO_4,_ 0.3 M NaCl, pH 8.0) and lysed by sonication (5 s on, 12 s off until clear). Cell debris and other insoluble substances were removed by centrifugation at 12,000 × *g* for 10 min at 4°C. The remaining supernatant was applied to nickel-nitrilotriacetic acid (Ni-NTA)-agarose solution (Qiagen) following the manufacturer’s recommendation. After washing with 400 μL washing buffer (1 M Na_2_HPO_4_, 1 M NaH_2_PO_4,_ 0.3 M NaCl) containing serial concentrations of imidazole (100/200 mM imidazole), the bound NrtR protein was eluted with 400 μL elution buffer (1 M Na_2_HPO_4_, 1 M NaH_2_PO_4_, 0.3 M NaCl, 400 mM imidazole). The purified protein was examined by SDS-PAGE.

### Electrophoretic mobility shift assays (EMSAs).

The electrophoretic mobility shift assay (EMSA) was performed as previously described with minor modifications (56). Briefly, DNA fragments corresponding to the sequences upstream of *nadD2*, *tssA1*, *rsmZ*, and *rsmY* and a fragment from the *nrtR* coding sequence were amplified by PCR using specific primers (Table S2). DNA fragments (40 ng) were incubated with 0, 4, or 8 μM purified recombinant His-NrtR protein in an ice bath for 25 min in a 20 μL reaction system (10 mM Tris, 1 mM DTT, pH 7.5). Afterwards, the samples were loaded onto an 8% native polyacrylamide gel, which had been prerun for 1 h, and then electrophoresed in 1 × TBE buffer (Tris-borate-EDTA: 0.089 M Tris, 0.089 M boric acid, 0.002 M EDTA, pH 8.3) at 10 mA for about 1.5 h in ice bath. Then, the gel was stained in 1 × TBE containing 0.5 μg/mL ethidium bromide for 10 min, and the bands were visualized in a ChemiDoc XRS + molecular imager (Bio-Rad, CA, USA).

### Chromatin immunoprecipitation (ChIP)-Seq.

Chromatin immunoprecipitation assays were performed according to methods previously described by Wuhan IGENEBOOK Biotechnology Co. Ltd. (56). Briefly, 2 × 10^10^ cells of Δ*nrtR*/pUCP24-*nrtR*-Flag were collected and cross-linked with 1% formaldehyde for 20 min at 37°C. The crosslinking was then stopped by the addition of glycine at a final concentration of 125 mM. Afterwards, bacterial cells were centrifuged, washed twice with Tris buffer (150 mM NaCl, 20 mM Tris-HCl pH 7.5) containing a complete proteinase inhibitor cocktail (Roche), and then resuspended in 400 μL lysis buffer (50 mM Tris–HCl pH 8.0, 10 mM EDTA, 1% SDS, 1% Triton X-100, mini-protease inhibitor cocktail [Roche]) for 30 min. The chromatin DNA was purified and sonicated to obtain soluble sheared chromatin with an average DNA length of 200–500 bp (20s on with 30s interval, 15 cycles, Diagenode Bioruptor Pico). Two μL of chromatin was saved at −20°C as input DNA, and 100 μL of chromatin was used for immunoprecipitation with 5 μg of anti-Flag antibody (F7425-2MG, Sigma-Aldrich) at 4°C overnight. The next day, 30 μL of protein G magnetic beads were added, and the reaction samples were further incubated at 4°C for 3 h. The beads were then washed with a series of washing buffers: once with low salt washing buffer (20 mM Tris-HCl pH 8.1, 150 mM NaCl, 2 mM EDTA, 1% Triton X-100, 0.1% SDS); twice with LiCl washing buffer (10 mM Tris-HCl pH 8.1, 250 mM LiCl, 1 mM EDTA, 1% NP40, 1% deoxycholic acid); and twice with TE buffer (10 mM Tris-HCl pH 7.5, 1 mM EDTA). Bound material was then eluted from the beads in 300 μL of elution buffer (100 mM NaHCO_3_, 1% SDS), treated first with RNase A at a final concentration of 8 μg/mL for 6 h at 65°C and then with proteinase K at a final concentration of 345 μg/mL overnight at 45°C. Immunoprecipitated DNA was used to construct sequencing libraries following the protocol provided by the I NEXTFLEX ChIP-Seq Library Prep Kit for Illumina Sequencing (NOVA-5143 Bioo Scientific) and sequenced on Illumina Xten with the PE 150 method.

Low-quality reads were filtered out via Trimmomatic software (version 0.38). Totally, 61,879,614 and 58,249,840 clean reads were obtained from the input and ChIP samples, respectively. The clean reads were then mapped to the PAK genome with Bwa (v.0.7.15), allowing up to two mismatches. Samtools (v. 1.3.1) software was used to remove potential PCR duplicates. The software MACS2 (v. 2.1.1.20160309) was utilized to call peaks with default parameters (bandwidth, 300 bp/value, 0.05/model fold, 5, 50).

### Biofilm formation assay.

Overnight cultured bacteria were diluted 50-fold in LB broth, grown to an OD_600_ of 1.0, and then diluted to an OD_600_ of 0.025 in LB medium. Two hundred μL of the diluted bacterial suspension was incubated in each well of a 96-well plate at 37°C for 24 h. After that, each well was washed three times with H_2_O, stained with 0.25% crystal violet for 10 min, and then washed with H_2_O for three times. Two hundred μL of destaining solution was added into each well and incubated at room temperature for 10 min. Each sample was measured at a wavelength of 590 nm using a microplate reader Varioskan Flash (Thermo Scientific, Billerica, MA, USA).

### Data availability.

The RNAseq and ChIP-seq data have been deposited in the NCBI Short Read Archive (SRA) database with accession numbers PRJNA733704 and PRJNA733326.
