# Supplementary material for: NrtR Mediated Regulation of H1-T6SS in Pseudomonas aeruginosa
Source: Microbiol Spectr. 2022 Feb 23;10(1):e01858-21. doi: 10.1128/spectrum.01858-21 (PMC8865458; doi:10.1128/spectrum.01858-21)
Supplement: SUPPLEMENTAL FILE 1 — Supplemental material. Download SPECTRUM01858-21_Supp_1_seq12.pdf, PDF file, 1.1 MB [file spectrum01858-21_supp_1_seq12.pdf]

Fig. S1

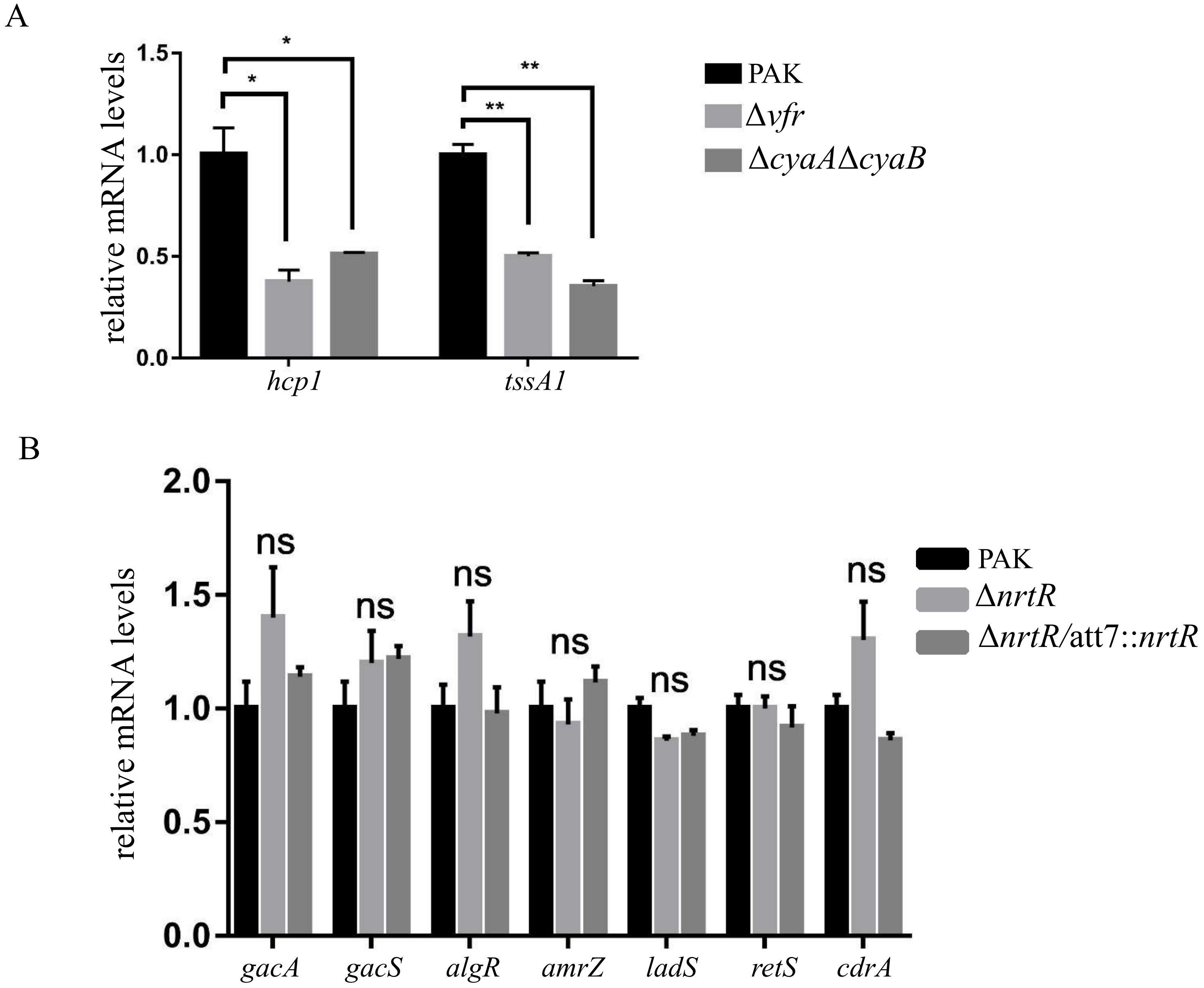

Fig. S1. The relative mRNA levels of the indicated genes in PAK, Δ*nrtR*, Δ*nrtR*/*att7*::*nrtR*, Δ*cyaA*Δ*cyaB* or Δ*vfr*. Total RNA was isolated from bacteria at an OD_{600} of 1.0, and the mRNA levels were examined by real-time qPCR using *rpsL* as an internal control. ns, not significant, *, *P*<0.05, **, *P*<0.01, compared to PAK by Student's *t* test.

Fig. S2

A

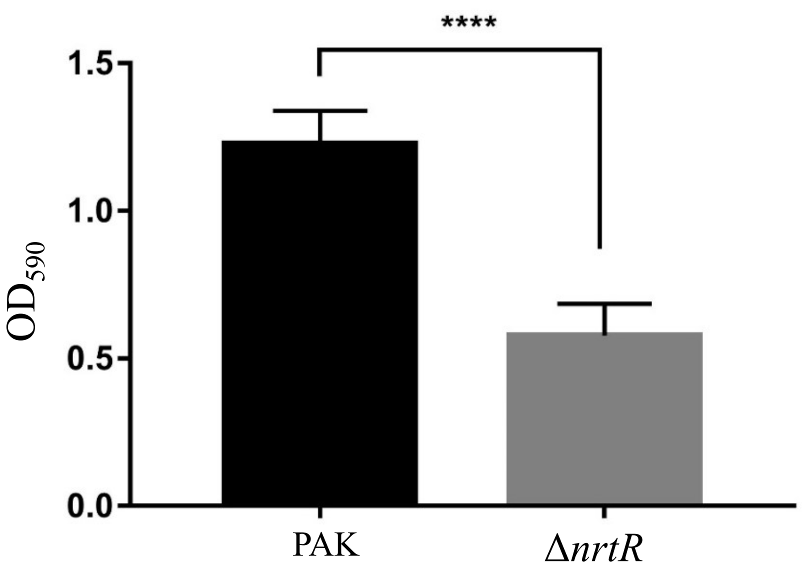

B

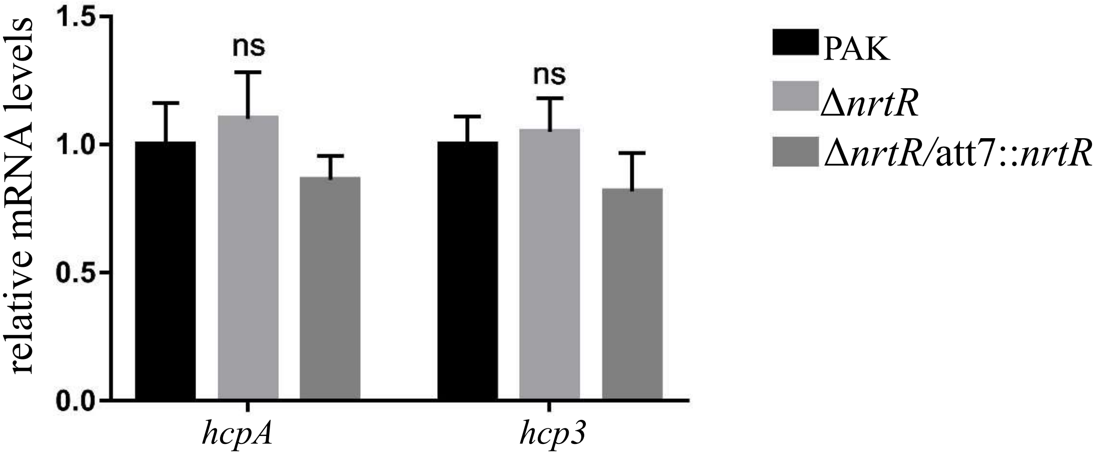

Fig. S2. (A) Biofilm formation by the PAK and Δ*nrtR* strains. PAK and Δ*nrtR* were grown in a 96-well plate for 24 h. The wells were washed with H_2O and stained with 2.5% crystal violet. The crystal violet was dissolved in destaining solution and measured at a wavelength of 590 nm. ****, $P<0.0001$, compared to PAK by Student's *t* test. **(B)** The relative mRNA levels of the indicated genes in PAK, Δ*nrtR*, and Δ*nrtR*/*att7*::*nrtR*. Total RNA was isolated from bacteria at an OD_{600} of 1.0, and the mRNA levels were examined by real-time qPCR using *rpsL* as an internal control. ns, not significant compared to PAK by Student's *t* test.

Table S1. Bacterial strains and plasmids used in this study.

Strain or plasmid	Description	Source or reference
**strains**		
E. coli strains		
DH5α	F^- ϕ 80d*lacZ*ΔM15 *endA1 recA1 hsdR17*($r_K^- m_K^+$) *supE44 thi-1 relA1* Δ(*lacZYA-argF*) *U169 gyrA96 deoR*	TransGen
S17-1	RP4-2 Tc::Mu Km::Tn*7* Tp^r Sm^r Pro Res^- Mod^+	Dr. Ramphal
BL21(DE3)	F^- *ompT hsdSB (rB$^-$, mB$^-$) gal dcm* (DE3)	invitrogen
***P. aeruginosa* strains**		
PAK	Wild type *P. aeruginosa* strain	(1)
Δ*nrtR*	PAK with *nrtR* deleted	(1)
Δ*nrtR*/att7::*hcp1-flag*	P_{hcp1}-*hcp1*-Flag integrated into the chromosome of Δ*nrtR* with mini-Tn7	This study
PAK/att7::*hcp1-flag*	P_{hcp1}-*hcp1*-Flag integrated into chromosome of PAK with mini-Tn7	This study
Δ*nrtR*/att7::*nrtR*	Δ*nrtR* with *nrtR* inserted into the chromosome with mini-Tn7 insertion	(1)
Δ*rsmY*Δ*rsmZ*	PAK with *rsmY* and *rsmZ* gene deleted	(2)
Δ*nrtR*Δ*rsmY*Δ*rsmZ*	Δ*nrtR* with *rsmY* and *rsmZ* gene deleted	This study
Δ*vfr*	PAK with *vfr* gene deleted	(1)
Δ*cyaA*Δ*cyaB*	PAK with *cyaA* and *cyaB* gene deleted	(1)
**Plasmids**		
pUCP20	Shuttle vector between *E. coli* and *P. aeruginosa*; Amp^r	(3)
pUCP24	Shuttle vector between *E. coli* and *P. aeruginosa*; Gm^r	(3)
pTNS3	Helper plasmid encoding Tn7 site-specific transposition pathway; Amp^r	(4)
pET28a	Expression vector, Kan^r	Novagen
E1553-*hcp1-flag*	*hcp1* with its promoter cloned into promoterless pUCP20	This study
pUCP20-*rsmA*	*rsmA* gene from PAK in pUCP20; Amp^r	This study
pUCP20-*rsmN*	*rsmN* gene from PAK in pUCP20; Amp^r	This study
pUCP24-*nrtR*	N terminal Flag-tagged *nrtR* from PAK in pUCP24; Gm^r	This study
pEX18-*rsmY*	*rsmY* gene deletion on pEX18Tc; Tc^r	(2)
pEX18-*rsmZ*	*rsmZ* gene deletion on pEX18Tc; Tc^r	(2)
p19-EGFP	EGFP gene cloned into pDN19*lacZ*Ω which was cut with *Bam*HI-*Hin*dIII, Tc^r	This study
P_{rsmY}-EGFP	*rsmY* promoter fused to P19-EGFP; Tc^r	This study
P_{rsmZ}-EGFP	*rsmZ* promoter fused to P19-EGFP; Tc^r	This study
P_{tssA1}-EGFP	*tssA1* promoter fused to P19-EGFP; Tc^r	This study

pUC18T-mini-Tn7T-Gm-*hcp1-flag*	Flag tagged *hcp1* on pUC18T-mini-Tn7T driven by *hcp1* promotor; Gm^r	(2)
pET28a-*nrtR*	*nrtR* of PAK cloned into pET28a, Kan^r	This study

1. Jin Y, Zhang M, Zhu F, Peng Q, Weng Y, Zhao Q, Liu C, Bai F, Cheng Z, Jin S, Wu W. 2019. NrtR Regulates the Type III Secretion System Through cAMP/Vfr Pathway in *Pseudomonas aeruginosa*. Front Microbiol 10:85.
2. Li K, Xu C, Jin Y, Sun Z, Liu C, Shi J, Chen G, Chen R, Jin S, Wu W. 2013. SuhB is a regulator of multiple virulence genes and essential for pathogenesis of *Pseudomonas aeruginosa*. mBio 4:e00419-13.
3. West SE, Schweizer HP, Dall C, Sample AK, Runyen-Janecky LJ. 1994. Construction of improved *Escherichia-Pseudomonas* shuttle vectors derived from pUC18/19 and sequence of the region required for their replication in *Pseudomonas aeruginosa*. Gene 148:81-6.
4. Choi KH, Schweizer HP. 2006. mini-Tn7 insertion in bacteria with single attTn7 sites: example *Pseudomonas aeruginosa*. Nat Protoc 1:153-61.

Table S2. Primers used in this study.

Primer[a]	Sequence 5’-3’[b]
pUCP-*rsmN*F	GGAATTCTGACTGGCAAGGACGCGGAACATGG
pUCP-*rsmN*R	CCCAAGCTTTCAGTGGTGGTGGTGGTGGTGGCCTTT CGGTGCCGTTTTCAAC
pUCP-*rsmA*F	GGAATTCTTCGCGTGAGGAGAAAGGAATGCTG
pUCP-*rsmA*R	CCCAAGCTTTTAGTGGTGGTGGTGGTGGTGATGGTT TGGCTCTTGATCTTTCTC
E1553-*hcp1-flag*F	GCTCTAGAACACCGACTTCGCCGCCTTCATCGG
E1553-*hcp1-flag*R	CCCAAGCTTTCACTTGTCGTCATCGTCCTTGTAGTC GGCCTGCACGTTCTGGCGGATGTTCC
*nrtR-flag*F	CGGGATCCGATTACAAGGACGACGATGACAAGAGT TCAGCGGAAGTATTGGCC
*nrtR-flag*R	CCCAAGCTTTCAGGACGCCAGCAGGCTGCG
pET- *nrtR*F	CCCATATGAGTTCAGCGGAAGTATTGGCC
pET- *nrtR*R	CCCTCGAGTCAGGACGCCAGCAGGCTG
P_{Tn7R}	CACAGCATAACTGGACTGATTTC
P_{glmS}-down	GCACATCGGCGACGTGCTCTC
P_{rsmY}F	ATCGGTCTCGAATTCGGTGGCCACGTAGTTCGG
P_{rsmY}R	ATCGGTCTCGGATCCGGTTTGAAGATTACGCATCTC
P_{rsmZ}F	GGAATTCCGAGCTGCTGCAGGATGAC
P_{rsmZ}R	CGGGATCC CAGGAGT GATATTAGCGATTC
P_{tssA1}F	GGAATTCGCTCCGGGTCCGGCAGGAC
P_{tssA1}R	CGGGATCCGGTGACGATCTCCCTATCATC
Deletion test primer	
*rsmY*TF	CGGCGAGCGGAACTATTACA
*rsmY*TR	TGCTGGAAGGCGTGGTCTGA
*rsmZ*TF	AGCATCTGGAGCGCTGATAC
*rsmZ*TR	GCCAAAAACGCTCGGTGAAT
EMSA primer	
*tssA1*F	TGCTACTCCTTGCATTGCCAG

*tssA1*R	GGTGACGATCTCCCTATCATC
*rsmZ*F	CAGGAGTGATATTAGCGATTCCCAG
*rsmZ*R	CGGAAAACCTTAGACCCACTGAAG
*rsmY*F	GCTGGGAAGGCTCGCGATGATGAG
*rsmY*R	CCGTATTGTCTTTGGCGCTTCCTGC
*nadD2*F	AGAAAATACCTTCCACTGCGAA
*nadD2*R	GGAAGCCTCCGCTTGAC
*nrtR*F	TGTTGCTGATCCGCCGCGCCCAGGC
*nrtR*R	CCGCGTTGCCGACCGTGGCCACCTG
tssA1up	GTTGCATGACACCGCAGATC
tssA1down	CCTTCCTTGACAAGCCTTGC
qPCR primer	
*hcp1*F	AGGACCTGTCGTTCACCAA
*hcp1*R	ATAGTGCTTGCCGCTGGA
*gacS*F	GAGGAAATGCAGCACAAC
*gacS*R	GTTCTGGATCTCGATGGT
*gacA*F	CCTGATGATCGCCAACTG
*gacA*R	ATAGGTATTCACGGTCTTCG
*algR*F	CCAGCAATGGCGAAGAAGC
*algR*R	TCATGGGCCGTGCAGAAG
*amrZ*F	GGCGCTGCAAGACAATCT
*amrZ*R	GTTCTGCTGACGGTGGGT
*rpsL*F	GTAAGGTATGCCGTGTACG
*rpsL*R	CACTACGCTGTGCTCTTG
*ladS*F	ATCTACAACCTGTTCATCTT
*ladS*R	CCGAAGGAAGCGATATAG
*retS*F	GATACTCGACATCTCCAA
*retS*R	GAAGATATCCAGGCAGTC
*cdrA*F	ATGTGAATCCGACTCTGA

*cdrA*R	CGTTGAACTGACTGTTGA
*hcpA*F	CAAGGTCGAGATCCAGTGGT
*hcpA*R	TCCAGGTGATCTTGCGGTAG
*hcp3*F	ATGGATGCGATCATTCTC
*hcp3*R	GTTGTGGCTGTAGGACAT

a: F: forward; R, reverse; T: test; b: The underlines are the sites of restriction enzymes.

Table S3. Transcriptome analysis: differentially expressed genes via RNAseq without those listed in Table 1.

Table S4. Potential NrtR binding sites identified via ChIP-Seq analysis without those listed in Table 2.
